# Impact of Chronic Kidney Disease Stages 3–5 on Mortality and Morbidity Outcomes in Patients with Esophageal Cancer: A Propensity-Score-Matched Cohort Study

**DOI:** 10.3390/curroncol33080474

**Published:** 2026-08-12

**Authors:** Tsai-Lung Yang, Cheng-Hao Chang, Chung-Kuan Wu

**Affiliations:** 1 Division of Thoracic Surgery, Department of Surgery, Shin-Kong Wu Ho-Su Memorial Hospital, Taipei 111045, Taiwan; 2 Division of Nephrology, Department of Internal Medicine, Shin-Kong Wu Ho-Su Memorial Hospital, Taipei 111045, Taiwan; 3 Department of General Medicine, Shin-Kong Wu Ho-Su Memorial Hospital, Taipei 111045, Taiwan; 4 Division of Digital Informatics Management, Department of Digital Medicine, Shin-Kong Wu Ho-Su Memorial Hospital, Taipei 111045, Taiwan; 5 School of Medicine, Fu Jen Catholic University, New Taipei City 242062, Taiwan

**Keywords:** chronic kidney disease, esophageal cancer, mortality, blood transfusion, cardiovascular events

## Abstract

Chronic kidney disease (CKD) may reduce physiologic reserve among patients with esophageal cancer, but evidence beyond postoperative cohorts is limited. Using the TriNetX Global Collaborative Network, we studied adults with esophageal cancer diagnosed during 2010–2023. CKD stages 3–5 were defined by diagnostic codes plus estimated glomerular filtration rate < 60 mL/min/1.73 m^2^ within 6 months before or on the index date; dialysis-dependent patients were excluded. Non-CKD patients served as comparators. Prespecified outcomes from day 1 to up to 3 years included all-cause mortality, subsequent recorded metastatic diagnosis, pneumonia, sepsis, blood transfusion, and major adverse cardiovascular events. Propensity score matching produced 832 pairs. All-cause mortality was not significantly higher in the overall cohort with CKD stages 3–5 than in the non-CKD cohort (HR, 1.13; 95% CI, 0.98–1.31; *p* = 0.099), whereas CKD stages 4–5 were associated with higher mortality in the stage-specific analysis (HR, 1.46; 95% CI, 1.03–2.06; *p* = 0.031). CKD stages 3–5 were also associated with higher risks of blood transfusion (HR, 1.45; 95% CI, 1.04–2.01; *p* = 0.025) and MACEs (HR, 1.22; 95% CI, 1.02–1.47; *p* = 0.027), and with a lower risk of subsequent recorded metastatic diagnosis (HR, 0.80; 95% CI, 0.65–0.99; *p* = 0.036). These findings suggest that the associations of CKD with post-diagnostic outcomes varied by outcome type, with higher mortality observed in the separate CKD stages 4–5 analysis.

## Simple Summary

Patients with esophageal cancer often need intensive treatment and supportive care. Kidney disease may make treatment more difficult because it is linked to anemia, cardiovascular disease, and reduced physiologic reserve. In this large electronic health record study, we compared adults with esophageal cancer who had clinically recorded chronic kidney disease stages 3–5 with similar adults without chronic kidney disease. Kidney disease was not associated with uniformly worse outcomes; instead, it was linked mainly to higher rates of blood transfusion and cardiovascular events, while more advanced kidney disease was linked to higher mortality. These findings suggest that kidney function should be considered when planning supportive care and risk counseling for patients with esophageal cancer.

## 1. Introduction

Esophageal cancer remains one of the most lethal solid tumors worldwide, and outcomes after diagnosis are shaped not only by tumor biology and stage but also by treatment-related complications and the patient’s ability to tolerate multimodality treatment [1,2]. After esophagectomy, postoperative pulmonary complications, including pneumonia, are common and have been associated with substantial perioperative morbidity and poorer long-term outcomes [3]. Preoperative anemia and perioperative transfusion have been associated with reduced progression-free survival after esophagectomy, while preoperative anemia has also been associated with poorer overall survival [4]. Cardiac substructure dose has been associated with major cardiovascular events after definitive radiotherapy [5]. These observations underscore the relevance of host vulnerability and treatment-related morbidity to post-diagnostic outcomes beyond tumor burden alone.

Chronic kidney disease (CKD) is a particularly relevant candidate. CKD stages 3–5 are accompanied by anemia, chronic inflammation, altered drug handling, and substantial cardiovascular burden [6,7,8]. In broader oncologic and surgical populations, renal dysfunction has been associated with perioperative morbidity, increased transfusion requirements, and worse short-term outcomes [9,10]. Direct renal evidence in esophageal cancer is available but remains concentrated in surgically treated patients and short-term perioperative outcomes. A propensity score-matched study reported that pre-existing CKD was associated with increased 30-day mortality and morbidity after esophagectomy [11]. In a separate esophagectomy cohort, pretreatment renal dysfunction defined by reduced estimated glomerular filtration rate (eGFR) was associated with severe postoperative complications, although mortality and long-term survival did not differ significantly [12]. Renal insufficiency and reduced preoperative eGFR have also been identified as risk factors for anastomotic leakage after esophagectomy [13,14]. A study of postoperative acute kidney injury further supports the clinical relevance of renal vulnerability following esophagectomy, although this study evaluated a postoperative complication rather than pre-existing CKD [15]. Thus, existing evidence suggests that impaired preoperative kidney function may increase perioperative risk, but it is largely restricted to selected surgical cohorts and short-term outcomes. The association of pre-existing non-dialysis CKD stages 3–5 with broader post-diagnostic outcomes across the overall esophageal cancer population, and whether these associations differ according to CKD severity, remains insufficiently characterized.

Given this limited and largely procedure-specific evidence base, clarifying this question may help identify a subgroup of patients who require closer multidisciplinary assessment and supportive care during cancer treatment. We therefore conducted a retrospective propensity score-matched cohort study using the TriNetX Global Collaborative Network to compare adults with esophageal cancer and pre-existing CKD stages 3–5 with matched adults with esophageal cancer without CKD. We examined all-cause mortality, subsequent recorded metastatic diagnosis, pneumonia, sepsis, blood transfusion, and major adverse cardiovascular events (MACEs), and additionally performed stage-specific analyses for CKD stage 3 and stages 4–5. We hypothesized that pre-existing CKD stages 3–5 would be associated with a higher risk of adverse clinical outcomes after the diagnosis of esophageal cancer.

## 2. Materials and Methods

### 2.1. Data Sources

This study was conducted using the TriNetX research network, a global federated platform that enables retrospective research using routinely collected electronic health records from multiple healthcare organizations. Within this network, participating institutions retain their data locally, and investigators access de-identified aggregate results through a secure web-based interface rather than through direct transfer of patient-level records. The platform contains longitudinal clinical information, including demographic characteristics, diagnoses, procedures, medications, and laboratory results, and standardizes these data across sites to support consistent cohort identification and outcome ascertainment. This structure allows real-world analyses across diverse healthcare settings while maintaining patient privacy and institutional control of source data.

### 2.2. Ethics Statement

This study was approved by the Institutional Review Board (IRB) of Shin Kong Wu Ho-Su Memorial Hospital (approval code: 20240716R; approval date: 30 August 2024). The requirement for informed consent was waived because the analysis used de-identified data obtained through the TriNetX research platform.

### 2.3. Study Design and Study Population

**Figure 1:**
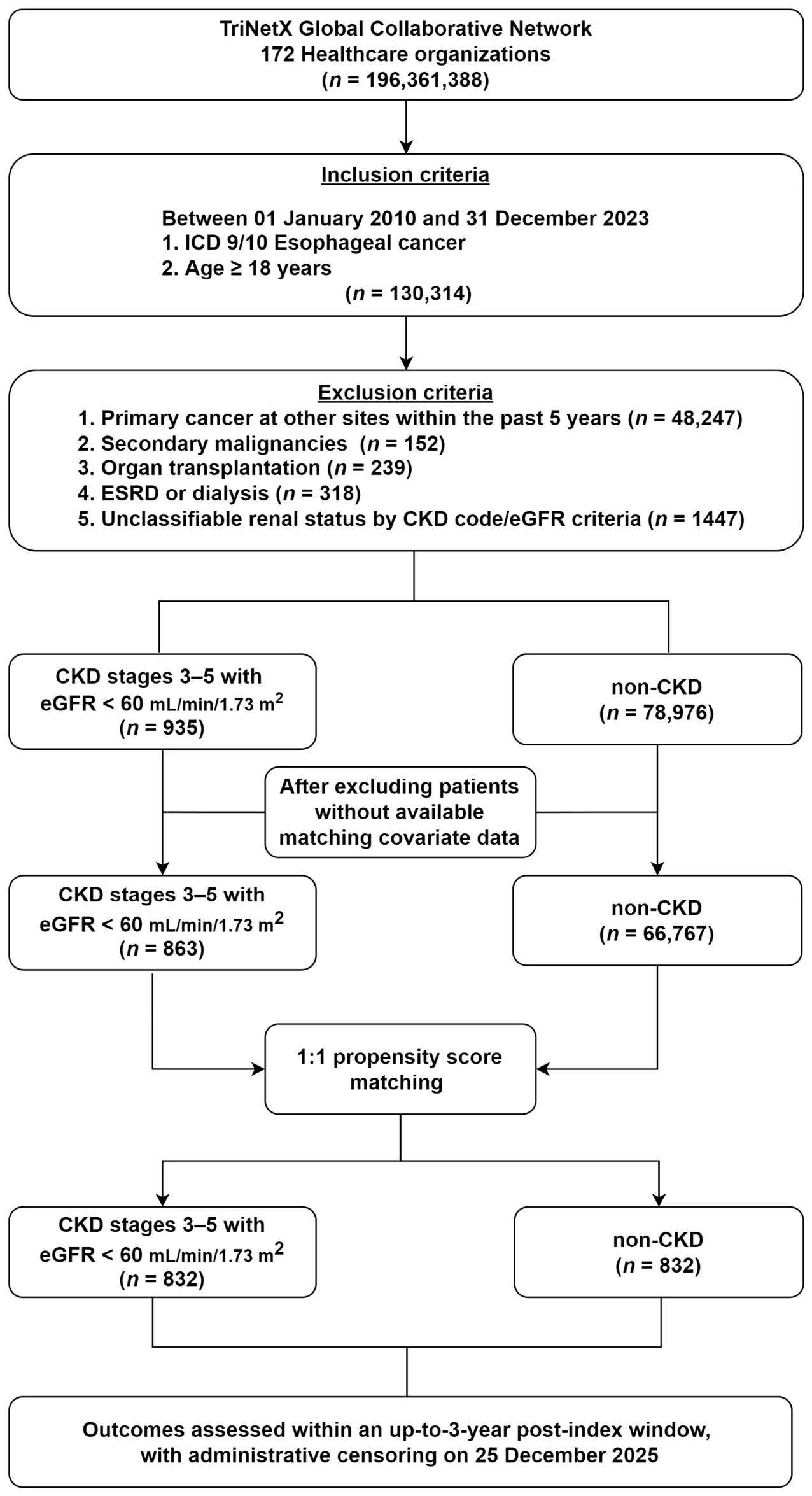
Study flowchart for cohort selection. Adults with esophageal cancer diagnosed between 1 January 2010 and 31 December 2023 were screened in the TriNetX Global Collaborative Network. After exclusion criteria were applied and patients with available matching covariates were identified, 1:1 propensity score matching yielded 832 patients in each cohort for the primary matched analysis. CKD, chronic kidney disease; eGFR, estimated glomerular filtration rate.

This retrospective cohort study used a propensity score-matched design to compare clinical outcomes between adults with esophageal cancer and pre-existing CKD stages 3–5 and those without CKD in the TriNetX Global Collaborative Network. Adults aged 18 years or older with a first recorded diagnosis of esophageal cancer between 1 January 2010 and 31 December 2023 were identified. The index date was defined as the date of the first recorded diagnosis of esophageal cancer during this period. Because cancer coding in routine practice may follow pathologic confirmation, diagnostic workup, or peri-diagnostic treatment encounters, the first recorded diagnosis code was used as the operational anchor for cohort entry. The exposure cohort comprised patients with CKD stages 3–5, defined by both a qualifying CKD diagnosis code and an eGFR < 60 mL/min/1.73 m^2^ within 6 months on or before the index date; patients meeting only one CKD criterion were not included in the comparative analyses. The 6-month laboratory window was selected to capture kidney function proximal to the esophageal cancer diagnosis while maintaining sufficient laboratory availability in the EHR. Because CKD is clinically defined by persistent kidney abnormality for more than 3 months, the diagnostic code was used to support chronicity, whereas the pre-index eGFR criterion was used to improve exposure specificity and severity classification. The comparison cohort included patients with esophageal cancer without CKD on or before the index date. Patients with diagnostic or procedure records indicating dialysis treatment or dialysis-dependent kidney disease before cohort entry were excluded. Additional exclusions were a recent history of primary cancer at other sites within 5 years before cohort entry, secondary malignancy on or before the index date, and organ transplantation. For the primary analysis, 1:1 propensity score matching (PSM) was performed after cohort construction using selected prespecified baseline covariates, and patients without an acceptable match were not retained in the matched analytic cohort. Separate stage-specific analyses were performed in independently constructed and independently propensity score-matched cohorts comparing CKD stage 3 with non-CKD and CKD stages 4–5 with non-CKD. Patients were followed from day 1 after the index date until the outcome of interest, death, 3 years of follow-up, or 25 December 2025, whichever occurred first. Figure 1 illustrates the cohort selection flow diagram. Detailed cohort definitions, CKD classification criteria, exclusion criteria, and outcome definitions are provided in Supplementary Methods S1.

### 2.4. Covariates

Baseline covariates were assessed during the year before the index date, and included age, sex, body mass index, and race; nicotine dependence and alcohol-related disorders; hypertension, diabetes mellitus, ischemic heart disease, heart failure, cerebrovascular disease, chronic obstructive pulmonary disease, and liver disease; and laboratory variables including hemoglobin, platelet count, blood urea nitrogen, creatinine, eGFR, sodium, potassium, chloride, calcium, phosphorus, magnesium, alanine aminotransferase, aspartate aminotransferase, albumin, and C-reactive protein. Because multimodality treatment is commonly used for locally advanced esophageal cancer, chemotherapy or chemoradiotherapy may precede definitive esophagectomy and surgical pathologic staging [2]. To capture the peri-diagnostic treatment context surrounding the first recorded esophageal cancer diagnosis code, treatment-related records within the baseline window included esophageal surgery, chemotherapy, and radiation therapy. These treatment-related variables were included as matching covariates; they did not define treatment groups and should not be interpreted as complete post-index treatment exposure. Baseline medication use was also recorded. Among the laboratory variables, hemoglobin and albumin were included in the primary PSM model. Other laboratory measures, including platelet count, blood urea nitrogen, creatinine, eGFR, electrolytes, mineral parameters, liver enzymes, and C-reactive protein, were retained for descriptive characterization but were not included in the primary matching algorithm. In particular, eGFR formed part of the operational CKD definition, while creatinine directly reflected kidney function. Detailed code lists and the prespecified variables selected for PSM are provided in Supplementary Methods S1.

### 2.5. Study Outcomes

The prespecified outcomes of interest were all-cause mortality, subsequent recorded metastatic diagnosis, pneumonia, sepsis, blood transfusion, and MACEs. Subsequent recorded metastatic diagnosis was defined as a new record of prespecified secondary malignant neoplasm codes during follow-up among patients without such codes at baseline. This endpoint was included as an electronic health record-based measure of newly documented metastatic disease after the index diagnosis. MACEs were defined as a composite of acute myocardial infarction, subsequent myocardial infarction, nontraumatic intracerebral hemorrhage, cerebral infarction, other or unspecified nontraumatic intracranial hemorrhage, transient ischemic attack, heart failure, or all-cause mortality. Outcomes were assessed from day 1 after the index date up to 3 years after cohort entry, with administrative censoring on 25 December 2025. Those whose index date occurred less than 3 years before the administrative end of data availability contributed their available follow-up time until the occurrence of an outcome event, death, or administrative censoring, as applicable. All outcomes were identified using prespecified diagnostic or procedure codes, with full definitions provided in Supplementary Methods S1.

### 2.6. Statistical Analysis

Baseline characteristics are presented as mean ± standard deviation for continuous variables and number (percentage) for categorical variables. To improve comparability between cohorts, 1:1 PSM was performed using logistic regression based on selected prespecified baseline covariates rather than all recorded baseline variables. The propensity score model included demographic characteristics, lifestyle-related variables, major comorbidities, hemoglobin, albumin, tumor histology, peri-diagnostic treatment-related variables, and baseline medication use. Covariate balance before and after matching was assessed using standardized mean differences, and an absolute standardized mean difference < 0.1 was considered to indicate acceptable balance [16]. For each outcome, patients who had the outcome before day 1 of the outcome window were excluded from the corresponding analysis, resulting in outcome-specific risk sets. Event-free survival was estimated using the Kaplan–Meier method and compared with the log-rank test. Hazard ratios and 95% confidence intervals were derived from Cox proportional hazards models, with the non-CKD cohort serving as the reference group. The primary matched analysis compared CKD stages 3–5 with non-CKD. Additional prespecified stage-specific analyses separately compared CKD stage 3 and CKD stages 4–5 with non-CKD. Exploratory subgroup analyses were performed across clinically relevant baseline strata. No formal adjustment for multiple comparisons was applied; therefore, subgroup findings were interpreted cautiously. All analyses were conducted using the TriNetX analytics platform, and custom R scripts (RStudio version 2025.05.1 + 513) were used for figure generation and supplementary data processing. All tests were two-sided, and *p* < 0.05 was considered statistically significant.

## 3. Results

Among 130,314 adults with esophageal cancer identified in the network during the study period, 935 met criteria for pre-existing CKD stages 3–5 with eGFR < 60 mL/min/1.73 m^2,^ and 78,976 had no CKD after application of the exclusion criteria. Of these, 863 patients with CKD stages 3–5 and 66,767 without CKD had available matching covariates and entered the pre-matching analytic cohort. PSM yielded 832 matched pairs for the primary analysis. After matching, the mean follow-up was 557.9 ± 428.7 days in the cohort with CKD stages 3–5 and 587.6 ± 431.1 days in the non-CKD cohort, with a median follow-up of 488 and 569 days, respectively.

### 3.1. Baseline Characteristics of the Matched Cohort

The baseline characteristics before and after matching are summarized in Table 1. In the matched cohort, the mean age was 71.31 ± 9.31 years in the group with CKD stages 3–5 and 71.18 ± 8.37 years in the non-CKD group, and the sex distribution was also well balanced. The mean body mass index was likewise similar between the two groups, at 27.99 ± 7.42 kg/m^2^ in the group with CKD stages 3–5 and 27.40 ± 7.03 kg/m^2^ in the non-CKD group. Lifestyle factors, including nicotine dependence and alcohol-related disorders, were similarly distributed after matching. The comorbidity burden was high in both groups, with hypertension being the most prevalent condition, followed by diabetes mellitus and ischemic heart disease, while the overall distribution of other major comorbidities was comparable. Most prespecified matching covariates presented in Table 1 achieved an absolute SMD < 0.1 after matching. Hemoglobin, which was included in the propensity score model, retained a moderate residual difference between the group with CKD stages 3–5 and the non-CKD group (11.41 ± 2.54 vs. 11.85 ± 2.60 g/dL; SMD, 0.172). The variables with post-matching SMDs > 0.2 were BUN, creatinine, eGFR, potassium, chloride, and phosphorus. These variables were not included in the propensity score model and are presented descriptively to characterize the renal and biochemical profiles of the cohorts. As expected, creatinine (1.83 ± 0.94 vs. 0.99 ± 0.50 mg/dL) and eGFR (41.64 ± 16.98 vs. 80.31 ± 30.09 mL/min/1.73 m^2^) remained substantially different because they directly reflected or operationally defined the CKD exposure. Treatment-related variables were also balanced after matching, with surgical procedures being more common than chemotherapy or radiation therapy in both groups. The chemotherapy and radiation therapy frequencies reflect only records captured during the pre-index baseline window and do not represent the total proportions of patients who received these treatments during the subsequent post-diagnostic course. In addition, baseline medication use was broadly comparable between cohorts after matching.

### 3.2. Associations of CKD Stages 3–5 with Clinical Outcomes

Clinical outcome analyses are summarized in Table 2. All-cause mortality did not differ significantly between the group with CKD stages 3–5 and the non-CKD group (HR, 1.13; 95% CI, 0.98–1.31; *p* = 0.099). Compared with the non-CKD group, CKD stages 3–5 were associated with higher risks of blood transfusion (HR, 1.45; 95% CI, 1.04–2.01; *p* = 0.025) and MACEs (HR, 1.22; 95% CI, 1.02–1.47; *p* = 0.027), as well as a lower risk of subsequent recorded metastatic diagnosis (HR, 0.80; 95% CI, 0.65–0.99; *p* = 0.036). No statistically significant differences were observed for pneumonia or sepsis between the two groups.

### 3.3. Stage-Specific Analyses by CKD Severity

**Figure 2:**
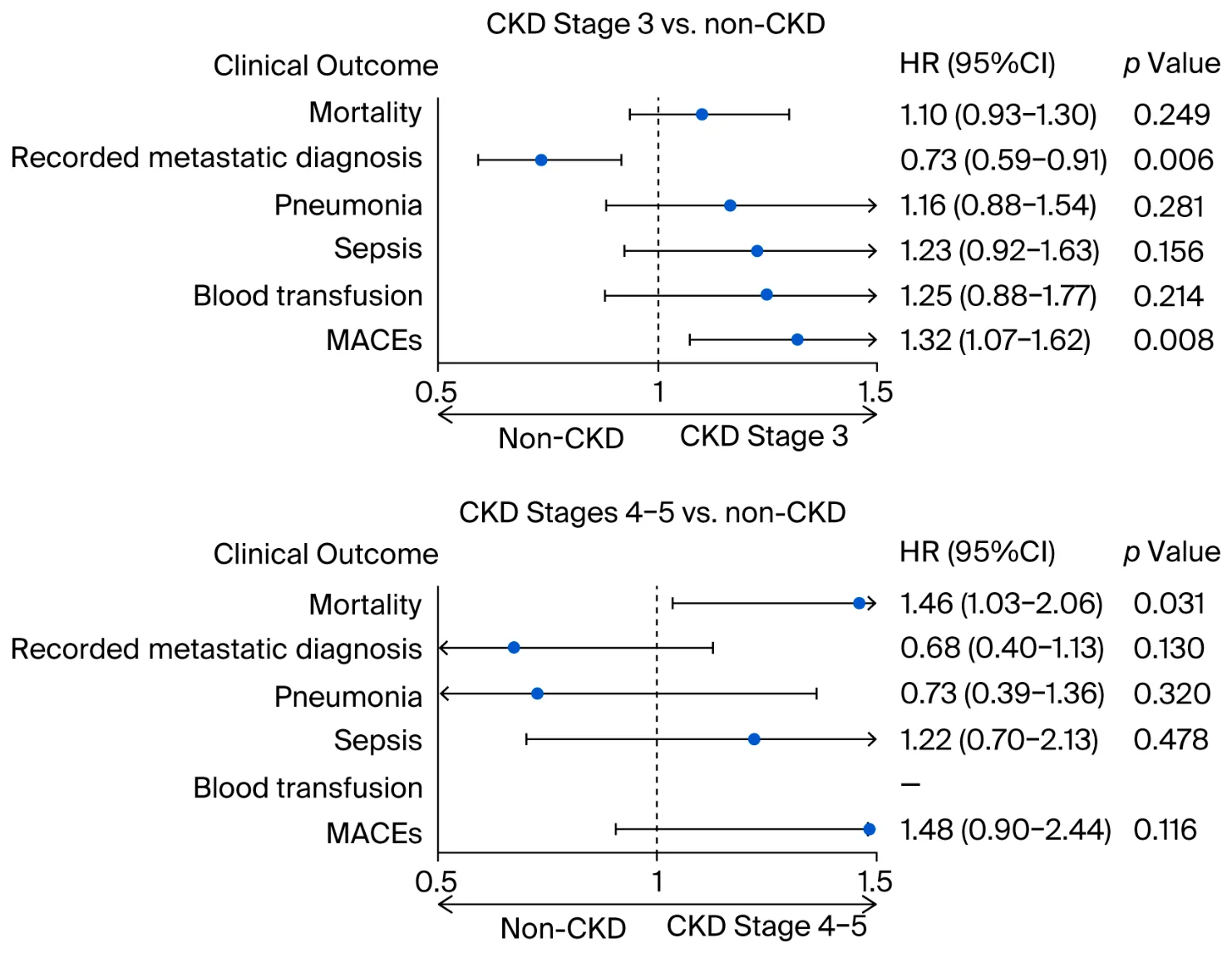
Stage-specific associations between CKD severity and clinical outcomes. Forest plots show hazard ratios and 95% confidence intervals for matched comparisons of CKD stage 3 versus non-CKD and CKD stages 4–5 versus non-CKD across the prespecified clinical outcomes. Hazard ratios > 1 indicate higher hazard in the CKD cohort. Blood transfusion was not estimable in the comparison of CKD stages 4–5, because of sparse events. Dots indicate point estimates, horizontal lines indicate 95% confidence intervals, and arrows indicate confidence intervals extending beyond the plotted range. CI, confidence interval; CKD, chronic kidney disease; HR, hazard ratio; MACEs, major adverse cardiovascular events.

Stage-specific associations are shown in Figure 2. All-cause mortality was not significantly different between CKD stage 3 and non-CKD, whereas CKD stages 4–5 were associated with higher all-cause mortality (HR, 1.46; 95% CI, 1.03–2.06; *p* = 0.031). In the CKD stage 3 analysis, CKD was associated with a lower risk of subsequent recorded metastatic diagnosis (HR, 0.73; 95% CI, 0.59–0.91; *p* = 0.006) and a higher risk of MACEs (HR, 1.32; 95% CI, 1.07–1.62; *p* = 0.008). In the analysis of CKD stages 4–5, associations with subsequent recorded metastatic diagnosis, pneumonia, sepsis, and MACEs did not reach statistical significance. No effect estimate was available for blood transfusion in the analysis of CKD stages 4–5, because of sparse events.

### 3.4. Exploratory Subgroup Analyses

Exploratory subgroup analyses are presented in Supplementary Figures S1–S6. Most subgroup point estimates were directionally consistent with the primary analyses, although several strata had wide confidence intervals because of limited event counts. Signals for higher pneumonia and sepsis risk appeared more evident among patients younger than 70 years (pneumonia: HR, 2.12; 95% CI, 1.07–4.19; *p* = 0.027; sepsis: HR, 2.33; 95% CI, 1.15–4.74; *p* = 0.016). Associations with blood transfusion also appeared stronger in some lower-comorbidity strata. Because these analyses were exploratory and no adjustment for multiple comparisons was applied, they should be interpreted as hypothesis-generating.

## 4. Discussion

In this propensity score-matched cohort of adults with esophageal cancer, all-cause mortality was not significantly higher in the overall cohort with CKD stages 3–5 than in the non-CKD cohort. In the stage-specific analysis, CKD stages 4–5 were associated with higher all-cause mortality, whereas CKD stage 3 was not. CKD stages 3–5 were also associated with higher risks of blood transfusion and MACEs. The lower risk of subsequent recorded metastatic diagnosis should be interpreted cautiously and should not be regarded as evidence of less aggressive tumor behavior. Overall, the observed associations differed according to the clinical outcome evaluated, although the observational design precludes causal inference.

The present findings should be considered in the context of the predominantly surgical evidence base. Previous esophagectomy studies have associated pre-existing CKD or reduced pretreatment eGFR with increased 30-day morbidity, severe postoperative complications, and anastomotic leakage [11,12,13,14]. Notably, mortality findings across these studies were not uniform, and most analyses were restricted to selected surgical cohorts and short-term perioperative endpoints. Our study extends this evidence by evaluating pre-existing non-dialysis CKD stages 3–5 across a broader esophageal cancer population, with outcomes up to 3 years post-diagnosis and separate analyses according to CKD severity.

In the present study, CKD stages 3–5 were associated with a higher hazard of recorded blood transfusion. Anemia is common in CKD and is clinically relevant during cancer treatment [6,7]. In large surgical oncology cohorts, CKD has been associated with higher transfusion rates and worse perioperative outcomes, and advanced renal dysfunction has been linked to an adjusted odds ratio of 2.74 for in-hospital mortality after elective cancer resection [9]. In esophageal cancer, preoperative anemia and perioperative transfusion have been associated with reduced progression-free survival, while transfusion after esophagectomy is influenced by multiple patient- and procedure-related factors [4,17]. Accordingly, the observed association may reflect several clinical factors, including baseline hematologic status and treatment context, and should not be interpreted as evidence of a specific CKD-mediated mechanism.

The MACEs finding represented another important morbidity outcome. CKD stages 3–5 were associated with a higher risk of the cardiovascular composite outcome in the primary matched analysis, and CKD stage 3 was also associated with the composite in its independently matched comparison. These findings are consistent with the established cardiovascular burden of CKD and with cohort evidence linking worsening kidney function and proteinuria to higher cardiovascular risk [8,18,19]. However, because MACEs were identified using the prespecified code-based composite definition, the observed association should be interpreted at the composite-outcome level and does not establish which individual cardiovascular component contributed to the result.

The stage-specific analyses showed different findings in the two independently matched comparisons. CKD stage 3 was associated with the cardiovascular composite outcome but not with higher all-cause mortality, whereas CKD stages 4–5 were associated with higher all-cause mortality. In a large community-based cohort, the risks of death and cardiovascular events increased progressively as estimated glomerular filtration rate declined below 60 mL/min/1.73 m^2^, with a substantially greater increase at more advanced levels of renal dysfunction [20]. A systematic review and meta-analysis of elective noncardiac surgery likewise demonstrated a graded relationship between CKD severity and postoperative death [10]. In oncology populations, CKD has also been associated with higher all-cause and cancer-specific mortality, and a severity-dependent relationship between renal impairment and cancer mortality has been reported [6,21]. However, because the stage-specific analyses used separately constructed and matched non-CKD comparator cohorts, differences in statistical significance should not be interpreted as a direct comparison between CKD stage 3 and CKD stages 4–5 or as proof of a graded severity–response relationship.

The lower hazard of subsequent recorded metastatic diagnosis in patients with CKD stages 3–5 should be interpreted cautiously. This endpoint captured newly documented metastatic disease in the electronic health record but did not include protocolized imaging, recurrence adjudication, or disease-free status and therefore should not be interpreted as disease-free survival. Because this outcome was based on coded metastatic diagnosis rather than centrally adjudicated cancer staging or imaging-confirmed progression, it may reflect documentation and ascertainment patterns rather than true tumor behavior. In multimorbid oncology populations, competing clinical events may substantially influence the observed incidence of individual outcomes, and failure to account for competing risks can bias event estimates [22,23]. In addition, coded diagnoses of metastasis have limited validity in administrative-style data, and death may act as a competing event for subsequent metastatic documentation [24]. Therefore, the lower risk of subsequent recorded metastatic diagnosis observed in our cohort is more appropriately interpreted as a finding potentially shaped by competing-risk and ascertainment effects, rather than as evidence of less aggressive tumor behavior.

The exploratory subgroup analyses showed larger point estimates for the association between CKD and blood transfusion in selected lower-comorbidity strata. Blood transfusion risk in esophageal cancer and surgical oncology is influenced by multiple patient and treatment-related factors [9,17]. However, because these analyses were not adjusted for multiple comparisons, some strata had limited event counts, and no formal interaction analysis was performed, the findings should be regarded as hypothesis-generating rather than as definitive evidence of subgroup-specific differences.

Our study had several strengths. First, it used a large global federated electronic health-record network, allowing evaluation of an understudied comorbidity in an esophageal cancer population not restricted to postoperative esophagectomy cohorts. Second, CKD stages 3–5 were defined using both diagnostic codes and an eGFR criterion, and patients with records indicating dialysis treatment or dialysis-dependent kidney disease were excluded to avoid mixing CKD stages 3–5 with dialysis-dependent kidney failure. Third, 1:1 PSM incorporated selected prespecified baseline covariates, including demographic characteristics, lifestyle-related factors, major comorbidities, selected laboratory variables, peri-diagnostic treatment-related variables, and baseline medication use, improving baseline comparability between cohorts. Finally, the study assessed multiple clinically relevant post-diagnostic outcomes and performed stage-specific analyses, allowing separate evaluation of associations in CKD stage 3 and CKD stages 4–5.

However, this study also has several limitations. First, its retrospective observational design precludes causal inference and remains susceptible to residual confounding despite propensity score matching. Although hemoglobin was included in the propensity score model, a moderate residual difference remained after matching and may have contributed to the observed association with blood transfusion. Second, detailed clinical or pathological TNM stage, formal performance-status measures, and treatment intent were not consistently available across participating healthcare organizations and therefore could not be reliably incorporated into the propensity score model. To reduce the potential influence of measured baseline differences, the propensity score model incorporated demographic and lifestyle-related variables, major comorbidities, hemoglobin, albumin, tumor histology when available, peri-diagnostic surgery, chemotherapy and radiotherapy records, and baseline medication use. These variables partially characterized baseline physiologic reserve, tumor type, comorbidity burden, and the treatment context surrounding the first recorded esophageal cancer diagnosis. Nevertheless, these measured covariates cannot fully substitute for direct TNM stage, formal performance status, or treatment intent. Residual confounding related to cancer burden and treatment selection therefore remains possible, particularly when interpreting all-cause mortality and subsequent recorded metastatic diagnosis. Third, CKD status and study outcomes were identified using coded electronic health record data and laboratory-based algorithms, which may have introduced misclassification. In particular, subsequent recorded metastatic diagnosis may have reflected delayed staging documentation rather than incident metastatic progression. Fourth, the composite cardiovascular outcome used in this study should be interpreted according to its prespecified component definitions, which may differ from conventional MACEs definitions used in other studies. Fifth, blood transfusion was identified from recorded procedure or clinical event codes, but the EHR data did not provide standardized information on transfusion indications, hemoglobin thresholds, symptoms, active bleeding status, perioperative context, or institutional transfusion protocols. Therefore, heterogeneity in transfusion practices across healthcare organizations may have influenced the observed transfusion outcome. Sixth, stage-specific analyses, particularly for CKD stages 4–5, had smaller sample sizes and sparse events for some outcomes, limiting precision and precluding estimation of blood transfusion risk in that subgroup. Finally, the findings reflect participating TriNetX healthcare organizations and may not be fully generalizable to other healthcare systems or practice settings.

## 5. Conclusions

In adults with esophageal cancer, CKD stages 3–5 were not associated with a statistically significant difference in all-cause mortality in the overall matched analysis, whereas CKD stages 4–5 were associated with higher all-cause mortality in the separate stage-specific analysis. CKD stages 3–5 were also associated with higher risks of blood transfusion and MACEs. These findings suggest that pre-existing CKD may be a relevant comorbidity when assessing post-diagnostic risk in esophageal cancer. Further studies incorporating detailed cancer stage, treatment intent, recurrence data, and complete treatment exposure are needed to confirm these associations.

## Figures and Tables

**Table 1:** Baseline characteristics of patients with CKD stages 3–5 and non-CKD patients.

	Before Matching			After Matching		
	CKD Stages 3–5 (*n* = 863)	Non-CKD (*n* = 66,767)	SMD	CKD Stages 3–5 (*n* = 832)	Non-CKD (*n* = 832)	SMD
Age (years), mean ± SD	71.51 ± 9.28	63.74 ± 10.5	0.784	71.31 ± 9.31	71.18 ± 8.37	0.016
Gender (male), n (%)	670 (77.64)	52,578 (78.75)	0.027	647 (77.76)	641 (77.04)	0.017
BMI (kg/m^2^), mean ± SD	28.06 ± 7.43	26.58 ± 6.57	0.211	27.99 ± 7.42	27.4 ± 7.03	0.081
White	640 (74.16)	37,129 (55.61)	0.396	619 (74.40)	626 (75.24)	0.019
Black or African American	104 (12.05)	3269 (4.90)	0.259	97 (11.66)	108 (12.98)	0.040
Asian	26 (3.01)	3416 (5.12)	0.107	26 (3.13)	18 (2.16)	0.060
Other race	19 (2.20)	3687 (5.52)	0.173	16 (1.92)	14 (1.68)	0.018
Lifestyles, n (%)						
Nicotine dependence	148 (17.15)	3920 (5.87)	0.359	145 (17.43)	156 (18.75)	0.034
Alcohol related disorders	71 (8.23)	1505 (2.25)	0.270	70 (8.41)	76 (9.14)	0.025
Comorbidities, n (%)						
Hypertension	742 (85.98)	12,278 (18.39)	1.837	711 (85.46)	717 (86.18)	0.021
Diabetes mellitus	393 (45.54)	4894 (7.33)	0.961	366 (43.99)	366 (43.99)	0.000
Ischemic heart diseases	383 (44.38)	4271 (6.40)	0.970	357 (42.91)	339 (40.75)	0.044
Heart failure	295 (34.18)	1384 (2.07)	0.917	268 (32.21)	240 (28.85)	0.073
Cerebrovascular diseases	126 (14.60)	1402 (2.10)	0.464	118 (14.18)	110 (13.22)	0.028
COPD	189 (21.90)	3037 (4.55)	0.530	181 (21.76)	186 (22.36)	0.014
Liver diseases	107 (12.40)	2162 (3.24)	0.346	105 (12.62)	105 (12.62)	0.000
Laboratory, mean ± SD						
Hemoglobin (g/dL)	11.35 ± 2.53	12.51 ± 2.42	0.469	11.41 ± 2.54	11.85 ± 2.60	0.172
Platelets (10^3^/uL)	236.45 ± 93.35	263.35 ± 109.00	0.265	236.92 ± 94.26	253.74 ± 106.06	0.168
BUN (mg/dL)	32.73 ± 19.32	16.72 ± 10.71	1.025	32.52 ± 19.13	20.08 ± 15.11	0.722
Creatinine (mg/dL)	1.83 ± 0.94	1.04 ± 3.72	0.291	1.83 ± 0.94	0.99 ± 0.50	1.120
eGFR (mL/min/1.73 m^2^)	41.51 ± 16.93	86.98 ± 31.34	1.805	41.64 ± 16.98	80.31 ± 30.09	1.583
Na (mmol/L)	138.22 ± 4.66	138.15 ± 4.16	0.016	138.26 ± 4.67	138.06 ± 4.23	0.044
K (mmol/L)	4.28 ± 0.63	4.13 ± 0.53	0.270	4.29 ± 0.63	4.10 ± 0.56	0.318
Cl (mmol/L)	103.03 ± 5.44	101.95 ± 5.62	0.194	103.11 ± 5.39	101.80 ± 5.13	0.249
Ca (mg/dL)	9.09 ± 0.79	9.17 ± 0.75	0.098	9.11 ± 0.78	9.12 ± 0.77	0.004
P (mg/dL)	3.59 ± 0.90	3.38 ± 0.93	0.239	3.58 ± 0.84	3.23 ± 0.79	0.423
Mg (mg/dL)	1.96 ± 0.43	1.92 ± 0.33	0.118	1.95 ± 0.43	1.94 ± 0.36	0.040
ALT (U/L)	24.72 ± 49.80	28.65 ± 52.10	0.077	24.98 ± 50.74	24.36 ± 27.87	0.015
AST (U/L)	33.25 ± 130.69	32.16 ± 55.40	0.011	33.46 ± 133.33	29.34 ± 32.73	0.042
Albumin (g/dL)	3.59 ± 0.64	3.74 ± 0.66	0.231	3.60 ± 0.64	3.56 ± 0.68	0.062
CRP (mg/L)	44.22 ± 64.38	38.49 ± 58.84	0.093	43.75 ± 63.66	47.19 ± 68.62	0.052
Treatment, n (%)						
Surgical procedures	258 (29.90)	7226 (10.82)	0.488	245 (29.45)	263 (31.61)	0.047
Chemotherapy	45 (5.21)	2686 (4.02)	0.057	45 (5.41)	42 (5.05)	0.016
Radiation therapy	10 (1.16)	1087 (1.63)	0.040	10 (1.20)	10 (1.20)	0.000
Medications, n (%)						
ACEI	193 (22.36)	4285 (6.42)	0.467	186 (22.36)	179 (21.51)	0.020
ARB	163 (18.89)	2683 (4.02)	0.480	153 (18.39)	155 (18.63)	0.006
Beta-blockers	424 (49.13)	6441 (9.65)	0.962	398 (47.84)	388 (46.64)	0.024
Loop diuretics	294 (34.07)	2233 (3.34)	0.857	269 (32.33)	255 (30.65)	0.036
Thiazides diuretics	152 (17.61)	2486 (3.72)	0.462	138 (16.59)	136 (16.35)	0.006
Metformin	61 (7.07)	2066 (3.09)	0.182	61 (7.33)	61 (7.33)	0.000
Sulfonylureas	64 (7.42)	837 (1.25)	0.306	57 (6.85)	56 (6.73)	0.005
DPP-4 inhibitors	41 (4.75)	417 (0.63)	0.257	36 (4.33)	36 (4.33)	0.000
Insulin	245 (28.39)	2535 (3.80)	0.710	224 (26.92)	206 (24.76)	0.049
Antilipemics	421 (48.78)	7333 (10.98)	0.907	393 (47.24)	394 (47.36)	0.002
Antiplatelets	306 (35.46)	4408 (6.60)	0.757	287 (34.50)	269 (32.33)	0.046
Anticoagulants	406 (47.05)	6165 (9.23)	0.927	382 (45.91)	377 (45.31)	0.012
Bronchodilators	322 (37.31)	5092 (7.63)	0.761	299 (35.94)	306 (36.78)	0.017
Ipratropium	162 (18.77)	1745 (2.61)	0.542	146 (17.55)	149 (17.91)	0.009
Proton pump inhibitors	461 (53.42)	14,180 (21.24)	0.706	436 (52.40)	453 (54.45)	0.041

SMD, standardized mean difference; SD, standard deviation; BMI, body mass index; COPD, chronic obstructive pulmonary disease; eGFR, estimated glomerular filtration rate; CRP, C-reactive protein; ARB, angiotensin II receptor blocker; ACEI, angiotensin-converting-enzyme inhibitor; BUN, blood urea nitrogen; ALT, alanine aminotransferase; AST, aspartate aminotransferase; DPP-4, dipeptidyl peptidase-4.

**Table 2:** Up-to-3-year clinical outcomes and Cox proportional hazards analyses between the group with CKD stages 3–5 and the non-CKD group.

Clinical Outcomes	CKD Stages 3–5	Non-CKD	CKD Stages 3–5 vs. Non-CKD	
	Events (*n*)/ Event-Free Probability (%)	Events (*n*)/ Event-Free Probability (%)	HR (95% CI)	*p* Value
Mortality	363 (46.8)	334 (51.0)	1.13 (0.98–1.31)	0.099
Recorded Metastatic diagnosis	164 (73.7)	205 (66.6)	0.80 (0.65–0.99)	0.036
Pneumonia	116 (72.9)	132 (70.9)	0.95 (0.74–1.22)	0.684
Sepsis	120 (75.2)	113 (77.4)	1.14 (0.88–1.47)	0.329
Blood transfusion	85 (84.5)	62 (87.9)	1.45 (1.04–2.01)	0.025
MACEs	234 (36.3)	240 (42.9)	1.22 (1.02–1.47)	0.027

CKD, chronic kidney disease; MACEs, major adverse cardiovascular events; HR, hazard ratio; CI, confidence interval.

## Data Availability

Restrictions apply to the availability of these data. Data were obtained from the TriNetX Global Collaborative Network and are available only to authorized users with appropriate institutional access and data use agreements and with the permission of TriNetX. The authors are not permitted to publicly share patient-level data from TriNetX. Aggregate results supporting the findings of this study are included in the article and Supplementary Materials.

## Supplementary Materials

The following supporting information can be downloaded at: https://www.mdpi.com/article/10.3390/curroncol33080474/s1. Supplementary Methods S1: cohort definitions, outcomes and baseline covariates; Figure S1: subgroup analysis for all-cause mortality; Figure S2: subgroup analysis for subsequent recorded metastatic diagnosis; Figure S3: subgroup analysis for pneumonia; Figure S4: subgroup analysis for sepsis; Figure S5: subgroup analysis for blood transfusion; Figure S6: subgroup analysis for major adverse cardiovascular events.
