# Supplementary material for: Impact of Chronic Kidney Disease Stages 3–5 on Mortality and Morbidity Outcomes in Patients with Esophageal Cancer: A Propensity-Score-Matched Cohort Study
Source: Curr Oncol. 2026 Aug 12;33(8):474. doi: 10.3390/curroncol33080474 (PMC13510328; doi:10.3390/curroncol33080474)
Supplement: Supplementary file 1 [file curroncol-33-00474-s001.zip › curroncol-4399397-supplementary.pdf]

# Supplementary Online Content

**Methods S1. Cohort definitions, outcomes and baseline covariates** ............... 2
**1. Cohort definitions** ............... 2
**Query criteria for cohort 1 (Esophageal cancer patients with CKD)** 2
**Query criteria for cohort 2 (Esophageal cancer patients without CKD)** ............... 5
**2. Codes and definitions of baseline covariates, including variables selected for propensity score matching** ............... 7
**3. Codes for outcome definitions** ............... 12
**Figure S1.** ............... 14
**Figure S2.** ............... 15
**Figure S3.** ............... 16
**Figure S4.** ............... 17
**Figure S5.** ............... 18
**Figure S6.** ............... 19

## Methods S1. Cohort definitions, outcomes and baseline covariates

## 1. Cohort definitions

### Query criteria for cohort 1 (Esophageal cancer patients with CKD)

| | | | | |
|---|---|---|---|---|
| **Group 1** | | | | |
| | **Group 1A** | | | |
| | must have | diagnosis | ICD-10-CM:C15 / ICD-9-CM:150 | Malignant neoplasm of esophagus (at least 18 years old at event) |
| | date constraint | The terms in this group occurred between Jan 1, 2010 and Dec 31, 2023 | | |
| **Group 2** | | | | |
| | **Group 2A** | | | |
| | must have | diagnosis | ICD-10-CM:C15 / ICD-9-CM:150 | Malignant neoplasm of esophagus (at least 18 years old at event) |
| | date constraint | The terms in this group occurred between Jan 1, 2010 and Dec 31, 2023 | | |
| | event relationship | Any instance of Group 2B occurred within 6 months on or before the first instance of Group 2A | | |
| | **Group 2B** | | | |
| | must have | diagnosis | ICD-10-CM:N18.9 / N18.3 / N18.4 / N18.5 | Chronic kidney disease, unspecified; stage 3 (moderate); stage 4 (severe); stage 5 |
| | cannot have | procedure / diagnosis | 1012740 / Z99.2 / 90945 / N18.6 | Dialysis Services and Procedures; dependence on renal dialysis; dialysis procedure other than hemodialysis; end stage renal disease |
| **Group 3** | | | | |
| | **Group 3A** | | | |
| | must have | diagnosis | ICD-10-CM:C15 / ICD-9-CM:150 | Malignant neoplasm of esophagus (at least 18 years old at event) |
| | date constraint | The terms in this group occurred between Jan 1, 2010 and Dec 31, 2023 | | |
| | event relationship | Any instance of Group 3B occurred within 5 years on or before the first instance of Group 3A | | |
| | **Group 3B** | | | |

| | | | | |
|---|---|---|---|---|
| cannot have | | diagnosis | C00-C14 / C30-C39 / C40-C41 / C43-C44 / C45-C49 / C50 | Malignant neoplasms of lip, oral cavity and pharynx; respiratory and intrathoracic organs; bone and articular cartilage; melanoma and other malignant neoplasms of skin; mesothelial and soft tissue; breast |
| | or | diagnosis | C51-C58 / C60-C63 / C64-C68 / C69-C72 / C73-C75 / C76-C80 | Malignant neoplasms of female genital organs; male genital organs; urinary tract; eye, brain and other parts of central nervous system; thyroid and other endocrine glands; ill-defined, other secondary and unspecified sites |
| | or | diagnosis | C7A / C7B / C81-C96 | Malignant neuroendocrine tumors; secondary neuroendocrine tumors; malignant neoplasms of lymphoid, hematopoietic and related tissue |
| | or | diagnosis | C16 / C17 / C18 / C19 / C20 / C21 / C22 / C23 / C24 / C25 / C26 / ICD-9-CM:154 | Malignant neoplasm of stomach; small intestine; colon; rectosigmoid junction; rectum; anus and anal canal; liver and intrahepatic bile ducts; gallbladder; other and unspecified parts of biliary tract; pancreas; other and ill-defined digestive organs; rectum, rectosigmoid junction, and anus |
| **Group 4** | | | | |
| **Group 4A** | | | | |
| must have | | diagnosis | ICD-10-CM:C15 / ICD-9-CM:150 | Malignant neoplasm of esophagus (at least 18 years old at event) |
| date constraint | | The terms in this group occurred between Jan 1, 2010 and Dec 31, 2023 | | |

| | | | |
|---|---|---|---|
| event relationship | Any instance of Group 4B occurred on or before the first instance of Group 4A | | |
| **Group 4B** | | | |
| cannot have | diagnosis | C77 / C78 / C79 | Secondary and unspecified malignant neoplasm of lymph nodes; secondary malignant neoplasm of respiratory and digestive organs; secondary malignant neoplasm of other and unspecified sites |
| **Group 5** | | | |
| **Group 5A** | | | |
| must have | diagnosis | ICD-10-CM:C15 / ICD-9-CM:150 | Malignant neoplasm of esophagus (at least 18 years old at event) |
| date constraint | The terms in this group occurred between Jan 1, 2010 and Dec 31, 2023 | | |
| event relationship | Any instance of Group 5B occurred on or before the first instance of Group 5A | | |
| **Group 5B** | | | |
| cannot have | diagnosis | Z94 | Transplanted organ and tissue status |
| **Group 6** | | | |
| **Group 6A** | | | |
| must have | diagnosis | ICD-10-CM:C15 / ICD-9-CM:150 | Malignant neoplasm of esophagus (at least 18 years old at event) |
| date constraint | The terms in this group occurred between Jan 1, 2010 and Dec 31, 2023 | | |
| event relationship | Any instance of Group 6B occurred within 6 months on or before the first instance of Group 6A | | |
| **Group 6B** | | | |
| must have | laboratory | TNX Curated 8001 | Glomerular filtration rate/1.73 sq M.predicted [Volume Rate/Area] in Serum, Plasma or Blood by Creatinine-based formula (MDRD) (at most 59.99 mL/min/{1.73_m2}) |

**Query criteria for cohort 2 (Esophageal cancer patients without CKD)**

| | | | | |
|---|---|---|---|---|
| **Group 1** | | | | |
| **Group 1A** | | | | |
| must have | | diagnosis | ICD-10-CM:C15 / ICD-9-CM:150 | Malignant neoplasm of esophagus (at least 18 years old at event) |
| date constraint | | The terms in this group occurred between Jan 1, 2010 and Dec 31, 2023 | | |
| **Group 2** | | | | |
| **Group 2A** | | | | |
| must have | | diagnosis | ICD-10-CM:C15 / ICD-9-CM:150 | Malignant neoplasm of esophagus (at least 18 years old at event) |
| date constraint | | The terms in this group occurred between Jan 1, 2010 and Dec 31, 2023 | | |
| event relationship | | Any instance of Group 2B occurred on or before the first instance of Group 2A | | |
| **Group 2B** | | | | |
| cannot have | | diagnosis | N18 | Chronic kidney disease (CKD) |
| **Group 3** | | | | |
| **Group 3A** | | | | |
| must have | | diagnosis | ICD-10-CM:C15 / ICD-9-CM:150 | Malignant neoplasm of esophagus (at least 18 years old at event) |
| date constraint | | The terms in this group occurred between Jan 1, 2010 and Dec 31, 2023 | | |
| event relationship | | Any instance of Group 3B occurred on or before the first instance of Group 3A | | |
| **Group 3B** | | | | |
| cannot have | | diagnosis | C00-C14 / C30-C39 / C40-C41 / C43-C44 / C45-C49 / C50 | Malignant neoplasms of lip, oral cavity and pharynx; respiratory and intrathoracic organs; bone and articular cartilage; melanoma and other malignant neoplasms of skin; mesothelial and soft tissue; breast |
| | or | diagnosis | C51-C58 / C60-C63 / C64-C68 / C69-C72 / C73-C75 / C76-C80 | Malignant neoplasms of female genital organs; male genital organs; urinary tract; eye, brain |

| | | | | |
|---|---|---|---|---|
| | | | | and other parts of central nervous system; thyroid and other endocrine glands; ill-defined, other secondary and unspecified sites |
| | or | diagnosis | C7A / C7B / C81-C96 | Malignant neuroendocrine tumors; secondary neuroendocrine tumors; malignant neoplasms of lymphoid, hematopoietic and related tissue |
| | or | diagnosis | C16 / C17 / C18 / C19 / C20 / C21 / C22 / C23 / C24 / C25 / C26 / ICD-9-CM:154 | Malignant neoplasm of stomach; small intestine; colon; rectosigmoid junction; rectum; anus and anal canal; liver and intrahepatic bile ducts; gallbladder; other and unspecified parts of biliary tract; pancreas; other and ill-defined digestive organs; rectum, rectosigmoid junction, and anus |
| **Group 4** | | | | |
| **Group 4A** | | | | |
| must have | | diagnosis | ICD-10-CM:C15 / ICD-9-CM:150 | Malignant neoplasm of esophagus (at least 18 years old at event) |
| date constraint | | The terms in this group occurred between Jan 1, 2010 and Dec 31, 2023 | | |
| event relationship | | Any instance of Group 4B occurred on or before the first instance of Group 4A | | |
| **Group 4B** | | | | |
| cannot have | | diagnosis | C77 / C78 / C79 | Secondary and unspecified malignant neoplasm of lymph nodes; secondary malignant neoplasm of respiratory and digestive organs; secondary malignant neoplasm of other and unspecified sites |
| **Group 5** | | | | |

| | | | |
|---|---|---|---|
| **Group 5A** | | | |
| must have | diagnosis | ICD-10-CM:C15 / ICD-9-CM:150 | Malignant neoplasm of esophagus (at least 18 years old at event) |
| date constraint | The terms in this group occurred between Jan 1, 2010 and Dec 31, 2023 | | |
| event relationship | Any instance of Group 5B occurred on or before the first instance of Group 5A | | |
| **Group 5B** | | | |
| cannot have | procedure / diagnosis | 90945 / 1012740 / N18.6 / Z99.2 | Dialysis procedure other than hemodialysis; Dialysis Services and Procedures; end stage renal disease; dependence on renal dialysis |
| **Group 6** | | | |
| **Group 6A** | | | |
| must have | diagnosis | ICD-10-CM:C15 / ICD-9-CM:150 | Malignant neoplasm of esophagus (at least 18 years old at event) |
| date constraint | The terms in this group occurred between Jan 1, 2010 and Dec 31, 2023 | | |
| event relationship | Any instance of Group 6B occurred on or before the first instance of Group 6A | | |
| **Group 6B** | | | |
| cannot have | diagnosis | Z94 | Transplanted organ and tissue status |

Note: In the original TriNetX query syntax, “cannot have” indicated that a patient was excluded from the cohort if the listed diagnosis or procedure was recorded during the specified temporal window.

## 2. Codes and definitions of baseline covariates, including variables selected for propensity score matching

Baseline covariates listed below were extracted from the TriNetX baseline patient characteristics output for this study.

Variables marked with an asterisk (*) were included in the primary propensity score matching model. Variables without an asterisk were prespecified baseline covariates collected for descriptive characterization or supplementary analyses and were not included in the primary matching algorithm.

For laboratory variables, the percentage not measured/recorded after matching was derived as 100% minus the matched percentage of the cohort with an available measurement in the uploaded export.

| **Demographics** | |
|---|---|
| Code | Name |
| AI | Age at Index* |
| M | Male* |
| 2106-3 | White* |
| 2054-5 | Black or African American* |
| 2028-9 | Asian* |
| 2131-1 | Other Race* |
| 1002-5 | American Indian or Alaska Native* |
| 2076-8 | Native Hawaiian or Other Pacific Islander* |
| **Diagnosis** | |
| Code / System | Name |
| ICD-10-CM I10-I1A | Hypertensive diseases* |
| ICD-10-CM E08-E13 | Diabetes mellitus* |
| ICD-10-CM I20-I25 | Ischemic heart diseases* |
| ICD-10-CM I50 | Heart failure* |
| ICD-10-CM J44 | Other chronic obstructive pulmonary disease* |
| ICD-10-CM F17 | Nicotine dependence* |
| ICD-10-CM I60-I69 | Cerebrovascular diseases* |
| ICD-10-CM K70-K77 | Diseases of liver* |
| ICD-10-CM F10 | Alcohol related disorders* |
| **Procedures** | |

| Code / System | Name |
| --- | --- |
| CPT 1007214 | Surgical Procedures on the Esophagus* |
| CPT 1007223 | Total or near total esophagectomy, without thoracotomy* |
| CPT 43287 | Esophagectomy, distal two-thirds, with laparoscopic mobilization of the abdominal and lower mediastinal esophagus and proximal gastrectomy, with laparoscopic pyloric drainage procedure if performed, with separate thoracoscopic mobilization of the middle and upper mediastinal esophagus and thoracic esophagogastrostomy (ie, laparoscopic thoracoscopic esophagectomy, Ivor Lewis esophagectomy) * |
| ICD-10-PCS 0DT5 | Gastrointestinal System / Resection / Esophagus* |
| CPT 1007226 | Total or near total esophagectomy, with thoracotomy* |
| CPT 1007230 | Partial esophagectomy, distal two-thirds, with thoracotomy and separate abdominal incision, with or without proximal gastrectomy* |
| CPT 1007234 | Partial esophagectomy, thoracoabdominal or abdominal approach, with or without proximal gastrectomy* |
| SNOMED 45900003 | Esophagectomy* |
| CPT 43286 | Esophagectomy, total or near total, with laparoscopic mobilization of the abdominal and mediastinal esophagus and proximal gastrectomy, with laparoscopic pyloric drainage procedure if performed, with open cervical pharyngogastrostomy or esophagogastrostomy (ie, laparoscopic transhiatal esophagectomy) * |
| CPT 43288 | Esophagectomy, total or near total, with thoracoscopic mobilization of the upper, middle, and lower mediastinal esophagus, with separate laparoscopic proximal gastrectomy, with laparoscopic pyloric drainage procedure if performed, with open cervical pharyngogastrostomy or esophagogastrostomy (ie, thoracoscopic, laparoscopic and cervical incision esophagectomy, McKeown esophagectomy, tri-incisional esophagectomy) * |
| ICD-10-PCS 0DT1 | Gastrointestinal System / Resection / Esophagus, Upper* |
| ICD-10-PCS 0DT2 | Gastrointestinal System / Resection / Esophagus, Middle* |
| ICD-10-PCS 0DT3 | Gastrointestinal System / Resection / Esophagus, Lower* |
| ICD-10-PCS 0DT4 | Gastrointestinal System / Resection / Esophagogastric Junction* |

| | |
|---|---|
| TNX Curated 1002 | Chemotherapy* |
| TNX Curated 1001 | Radiation* |
| **Oncology** | |
| Code / System | Name |
| ICD-O 814-838 | Adenomas and adenocarcinomas* |
| ICD-O 805-808 | Squamous cell neoplasms* |
| **Medication** | |
| Code / System | Name |
| ATC A02BC | Proton pump inhibitors* |
| VA CV100 | Beta blockers/related* |
| VA CV350 | Antilipemic agents* |
| VA BL110 | Anticoagulants* |
| VA CV700 | Diuretics* |
| VA RE102 | Bronchodilators,sympathomimetic,inhalation* |
| VA BL117 | Platelet aggregation inhibitors* |
| VA CV702 | Loop diuretics* |
| VA HS501 | Insulin* |
| VA CV800 | Ace inhibitors* |
| VA CV805 | Angiotensin ii inhibitor* |
| RxNorm 7213 | Ipratropium* |
| VA CV701 | Thiazides/related diuretics* |
| RxNorm 6809 | Metformin* |
| ATC A10BB | Sulfonylureas* |
| ATC A10BH | Dipeptidyl peptidase 4 (dpp-4) inhibitors* |
| RxNorm 10438 | Theophylline* |
| RxNorm 34530 | Procaterol* |
| RxNorm 4333 | Fenoterol* |

| Laboratory data | | | |
|---|---|---|---|
| TNX Curated / source code | Name (unit) | Percentage not measured/recorded (After matching) | |
| | | CKD group | No CKD group |
| 8001 | Glomerular filtration rate/1.73 sq M.predicted [Volume Rate/Area] in Serum, Plasma or Blood by Creatinine-based formula (MDRD) | 0.00% | 9.50% |
| 9024 | Creatinine [Mass/volume] in Serum, Plasma or Blood | 1.68% | 9.74% |
| 9029 | Sodium [Moles/volume] in Serum, Plasma or Blood | 3.00% | 7.45% |
| 9028 | Potassium [Moles/volume] in Serum, Plasma or Blood | 4.45% | 7.45% |
| 9025 | Glucose [Mass/volume] in Serum, Plasma or Blood | 6.01% | 10.34% |
| 9020 | Platelets [#/volume] in Blood | 6.85% | 7.09% |
| 9014 | Hemoglobin [Mass/volume] in Blood* | 6.97% | 5.17% |
| 9030 | Urea nitrogen [Mass/volume] in Serum, Plasma or Blood | 7.57% | 14.54% |
| 9022 | Calcium [Mass/volume] in Serum, Plasma or Blood | 8.05% | 13.10% |
| 9023 | Chloride [Moles/volume] in Serum, Plasma or Blood | 9.01% | 12.50% |
| 9044 | Alanine aminotransferase [Enzymatic activity/volume] in Serum, Plasma or Blood | 14.66% | 15.86% |
| 9045 | Albumin [Mass/volume] in Serum, Plasma or Blood* | 15.62% | 18.75% |
| 9047 | Aspartate aminotransferase [Enzymatic activity/volume] in Serum or Plasma | 16.95% | 18.15% |
| 9083 | BMI* | 31.61% | 30.17% |
| 9032 | INR in Plasma or Blood | 48.20% | 46.39% |

| | | | |
|---|---|---|---|
| 9026 | Magnesium [Mass/volume] in Serum, Plasma or Blood | 55.53% | 59.74% |
| 9027 | Phosphate [Mass/volume] in Serum, Plasma or Blood | 58.17% | 71.88% |
| 9063 | C reactive protein [Mass/volume] in Serum, Plasma or Blood | 83.05% | 86.66% |

## 3. Codes for outcome definitions

Table below outlines the definitions for each outcome. For outcome definitions consisting of more than one term, at least one listed code qualified as the outcome.

| **Primary outcome** | | |
|---|---|---|
| **Mortality** | | |
| **Status** | Deceased | Deceased |
| **Secondary outcomes** | | |
| **Subsequent recorded metastatic diagnosis** | | |
| **Diagnosis** | ICD-10-CM:C77 | Secondary and unspecified malignant neoplasm of lymph nodes |
| **Diagnosis** | ICD-10-CM:C78 | Secondary malignant neoplasm of respiratory and digestive organs |
| **Diagnosis** | ICD-10-CM:C79 | Secondary malignant neoplasm of other and unspecified sites |
| **Pneumonia** | | |
| **Diagnosis** | ICD-10-CM:J18 | Pneumonia, unspecified organism |
| **Diagnosis** | ICD-10-CM:J16 | Pneumonia due to other infectious organisms, not elsewhere classified |
| **Diagnosis** | ICD-10-CM:J13 | Pneumonia due to Streptococcus pneumoniae |
| **Diagnosis** | ICD-10-CM:J17 | Pneumonia in diseases classified elsewhere |
| **Diagnosis** | ICD-10-CM:J14 | Pneumonia due to Hemophilus influenzae |
| **Diagnosis** | ICD-10-CM:J15 | Bacterial pneumonia, not elsewhere classified |
| **Sepsis** | | |
| **Diagnosis** | ICD-10-CM:A40 | Streptococcal sepsis |
| **Diagnosis** | ICD-10-CM:A41 | Other sepsis |

| | | |
|---|---|---|
| **Blood transfusion** | | |
| **Procedure** | ICD-10-PCS:30233H1 | Transfusion of Nonautologous Whole Blood into Peripheral Vein, Percutaneous Approach |
| **Procedure** | ICD-10-PCS:30233N1 | Transfusion of Nonautologous Red Blood Cells into Peripheral Vein, Percutaneous Approach |
| **Procedure** | ICD-9-CM:99.0 | Transfusion of blood and blood components |
| **Procedure** | ICD-10-PCS:30243H1 | Transfusion of Nonautologous Whole Blood into Central Vein, Percutaneous Approach |
| **Procedure** | ICD-10-PCS:30243N1 | Transfusion of Nonautologous Red Blood Cells into Central Vein, Percutaneous Approach |
| **MACEs** | | |
| **Diagnosis** | ICD-10-CM:I21 | Acute myocardial infarction |
| **Diagnosis** | ICD-10-CM:I22 | Subsequent ST elevation (STEMI) and non-ST elevation (NSTEMI) myocardial infarction |
| **Diagnosis** | ICD-10-CM:I61 | Nontraumatic intracerebral hemorrhage |
| **Diagnosis** | ICD-10-CM:I63 | Cerebral infarction |
| **Diagnosis** | ICD-10-CM:I62 | Other and unspecified nontraumatic intracranial hemorrhage |
| **Diagnosis** | ICD-10-CM:G45.9 | Transient cerebral ischemic attack, unspecified |
| **Status** | Deceased | Deceased |
| **Diagnosis** | ICD-10-CM:I50 | Heart failure |

## Figure S1.

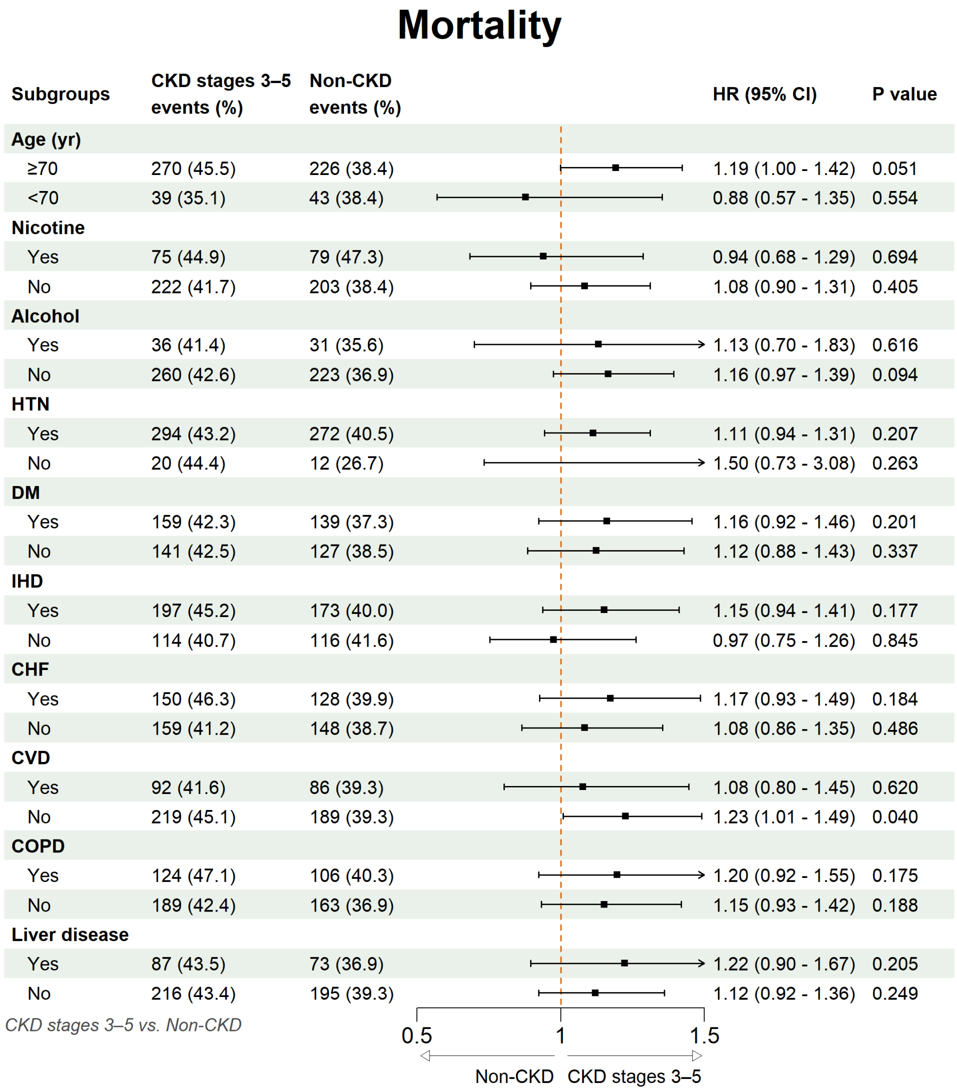


**Figure S1. Subgroup analysis for all-cause mortality.** Forest plot of hazard ratios (HRs) with 95% CI for all-cause mortality in matched esophageal cancer patients with CKD stages 3–5 versus those without CKD. Subgroups were stratified by age, sex, nicotine dependence, alcohol related disorders, hypertensive diseases, ischemic heart diseases, heart failure, cerebrovascular diseases, chronic obstructive pulmonary disease, and liver diseases. HR > 1 indicates higher hazard in the CKD cohort.

**Figure S2.**

## Metastasis

| Subgroups | CKD stages 3–5 events (%) | Non-CKD events (%) | HR (95% CI) | P value |
|---|---|---|---|---|
| **Age (yr)** | | | | |
| ≥70 | 109 (18.3) | 142 (23.9) | 0.74 (0.57 - 0.95) | 0.017 |
| <70 | 35 (31.2) | 28 (25.0) | 1.25 (0.76 - 2.06) | 0.369 |
| **Nicotine** | | | | |
| Yes | 39 (23.4) | 48 (28.7) | 0.77 (0.50 - 1.17) | 0.215 |
| No | 106 (19.9) | 130 (24.3) | 0.78 (0.60 - 1.01) | 0.058 |
| **Alcohol** | | | | |
| Yes | 17 (19.5) | 16 (18.4) | 1.01 (0.51 - 2.00) | 0.982 |
| No | 121 (19.7) | 147 (23.9) | 0.80 (0.63 - 1.02) | 0.073 |
| **HTN** | | | | |
| Yes | 136 (19.9) | 179 (26.2) | 0.75 (0.60 - 0.94) | 0.011 |
| No | 11 (23.9) | 12 (26.1) | 0.75 (0.33 - 1.71) | 0.493 |
| **DM** | | | | |
| Yes | 83 (22.0) | 105 (27.9) | 0.77 (0.58 - 1.02) | 0.072 |
| No | 62 (18.6) | 80 (24.0) | 0.75 (0.54 - 1.05) | 0.089 |
| **IHD** | | | | |
| Yes | 90 (20.6) | 107 (24.5) | 0.82 (0.62 - 1.08) | 0.158 |
| No | 56 (19.9) | 80 (28.4) | 0.68 (0.48 - 0.95) | 0.024 |
| **CHF** | | | | |
| Yes | 63 (19.4) | 71 (21.8) | 0.87 (0.62 - 1.22) | 0.424 |
| No | 82 (21.2) | 99 (25.6) | 0.80 (0.60 - 1.08) | 0.146 |
| **CVD** | | | | |
| Yes | 40 (18.0) | 57 (25.7) | 0.65 (0.43 - 0.97) | 0.033 |
| No | 105 (21.6) | 114 (23.4) | 0.94 (0.72 - 1.23) | 0.654 |
| **COPD** | | | | |
| Yes | 51 (19.3) | 77 (29.2) | 0.62 (0.44 - 0.89) | 0.008 |
| No | 97 (21.7) | 116 (25.9) | 0.81 (0.62 - 1.06) | 0.129 |
| **Liver disease** | | | | |
| Yes | 50 (25.0) | 64 (32.0) | 0.74 (0.51 - 1.07) | 0.110 |
| No | 94 (18.8) | 101 (20.2) | 0.91 (0.68 - 1.20) | 0.491 |

*CKD stages 3–5 vs. Non-CKD*

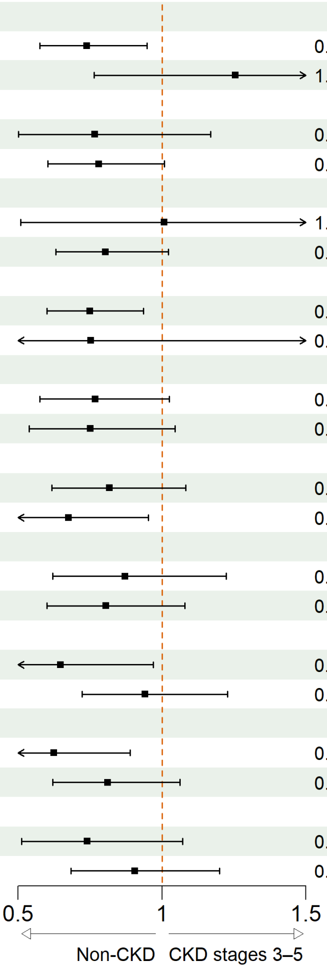


**Figure S2. Subgroup analysis for subsequent recorded metastatic diagnosis.** Forest plot of hazard ratios (HRs) with 95% CI for subsequent recorded metastatic diagnosis in matched esophageal cancer patients with CKD stages 3–5 versus those without CKD. Subsequent recorded metastatic diagnosis was defined by secondary malignant neoplasms of lymph nodes, respiratory or digestive organs, or other and unspecified sites. HR > 1 indicates higher hazard in the CKD cohort.

## Figure S3.

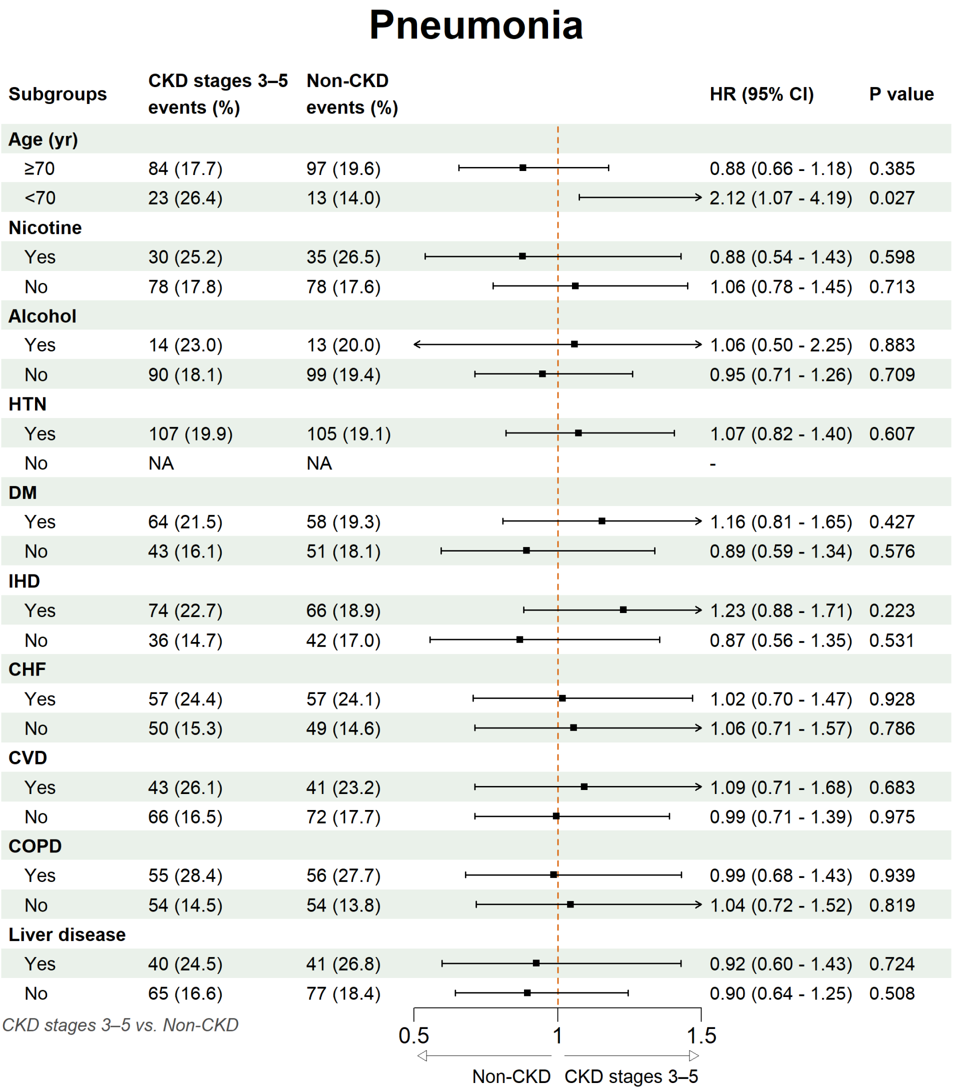


**Figure S3. Subgroup analysis for pneumonia.** Forest plot of hazard ratios (HRs) with 95% CI for pneumonia in matched esophageal cancer patients with CKD stages 3–5 versus those without CKD. Pneumonia was defined by the prespecified ICD-10-CM codes listed in Methods S1. HR > 1 indicates higher hazard in the CKD cohort.

## Figure S4.

# Sepsis

| Subgroups | CKD stages 3–5 events (%) | Non-CKD events (%) | HR (95% CI) | P value |
|---|---|---|---|---|
| **Age (yr)** | | | | |
| ≥70 | 77 (15.0) | 83 (15.4) | 0.94 (0.69 - 1.29) | 0.719 |
| <70 | 25 (25.8) | 11 (11.2) | 2.33 (1.15 - 4.74) | 0.016 |
| **Nicotine** | | | | |
| Yes | 30 (21.4) | 26 (17.9) | 1.13 (0.67 - 1.92) | 0.639 |
| No | 68 (14.7) | 72 (14.7) | 0.99 (0.71 - 1.38) | 0.944 |
| **Alcohol** | | | | |
| Yes | 13 (17.8) | 11 (15.7) | 1.07 (0.48 - 2.40) | 0.863 |
| No | 87 (16.4) | 73 (12.9) | 1.26 (0.92 - 1.72) | 0.150 |
| **HTN** | | | | |
| Yes | 102 (17.4) | 98 (15.8) | 1.11 (0.84 - 1.47) | 0.447 |
| No | NA | NA | - | |
| **DM** | | | | |
| Yes | 62 (19.1) | 61 (18.2) | 1.05 (0.73 - 1.49) | 0.800 |
| No | 38 (13.3) | 37 (11.9) | 1.13 (0.72 - 1.78) | 0.589 |
| **IHD** | | | | |
| Yes | 74 (20.1) | 67 (17.2) | 1.15 (0.82 - 1.59) | 0.420 |
| No | 31 (12.5) | 28 (10.4) | 1.16 (0.70 - 1.94) | 0.567 |
| **CHF** | | | | |
| Yes | 58 (21.5) | 57 (20.5) | 1.03 (0.72 - 1.49) | 0.868 |
| No | 45 (13.2) | 43 (12.1) | 1.08 (0.71 - 1.64) | 0.715 |
| **CVD** | | | | |
| Yes | 35 (18.2) | 30 (14.6) | 1.22 (0.75 - 1.99) | 0.420 |
| No | 67 (16.0) | 70 (15.8) | 1.04 (0.74 - 1.46) | 0.815 |
| **COPD** | | | | |
| Yes | 50 (21.9) | 49 (21.0) | 1.00 (0.68 - 1.49) | 0.996 |
| No | 56 (14.5) | 55 (13.5) | 1.06 (0.73 - 1.54) | 0.760 |
| **Liver disease** | | | | |
| Yes | 37 (21.5) | 40 (23.0) | 0.94 (0.60 - 1.48) | 0.800 |
| No | 65 (15.1) | 58 (12.7) | 1.17 (0.82 - 1.67) | 0.378 |

*CKD stages 3–5 vs. Non-CKD*

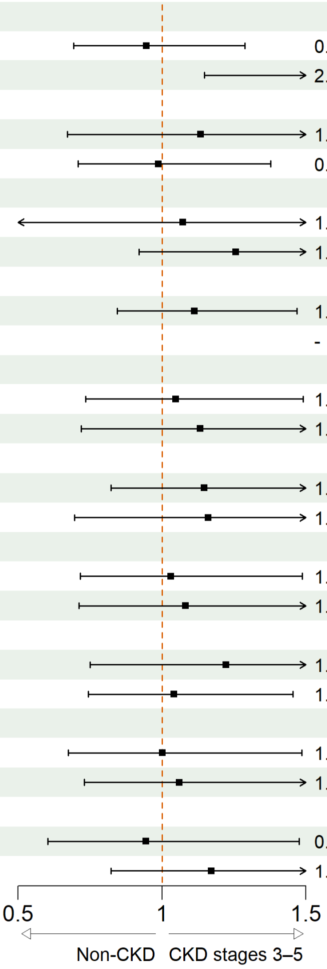


**Figure S4. Subgroup analysis for sepsis.** Forest plot of hazard ratios (HRs) with 95% CI for sepsis in matched esophageal cancer patients with CKD stages 3–5 versus those without CKD. Sepsis was defined by streptococcal sepsis or other sepsis. HR > 1 indicates higher hazard in the CKD cohort.

## Figure S5.

### Blood transfusion

| Subgroups | CKD stages 3–5 events (%) | Non-CKD events (%) | HR (95% CI) | P value |
|---|---|---|---|---|
| **Age (yr)** | | | | |
| ≥70 | 50 (9.1) | 45 (7.9) | 1.13 (0.76 - 1.70) | 0.543 |
| <70 | NA | NA | - | |
| **Nicotine** | | | | |
| Yes | 21 (13.8) | 14 (9.1) | 1.49 (0.76 - 2.92) | 0.249 |
| No | 47 (9.4) | 34 (6.6) | 1.43 (0.92 - 2.22) | 0.113 |
| **Alcohol** | | | | |
| Yes | NA | NA | - | |
| No | 57 (9.9) | 35 (6.0) | 1.69 (1.11 - 2.58) | 0.013 |
| **HTN** | | | | |
| Yes | 68 (10.8) | 63 (9.7) | 1.13 (0.80 - 1.59) | 0.489 |
| No | NA | NA | - | |
| **DM** | | | | |
| Yes | 44 (12.7) | 40 (11.2) | 1.14 (0.74 - 1.74) | 0.563 |
| No | 25 (7.9) | 14 (4.3) | 1.89 (0.98 - 3.63) | 0.053 |
| **IHD** | | | | |
| Yes | 46 (11.6) | 39 (9.5) | 1.22 (0.80 - 1.87) | 0.363 |
| No | 25 (9.2) | 17 (6.3) | 1.49 (0.81 - 2.76) | 0.200 |
| **CHF** | | | | |
| Yes | 29 (9.7) | 38 (12.8) | 0.73 (0.45 - 1.19) | 0.205 |
| No | 39 (10.7) | 20 (5.4) | 2.04 (1.19 - 3.50) | 0.008 |
| **CVD** | | | | |
| Yes | 27 (13.6) | 12 (6.0) | 2.30 (1.16 - 4.54) | 0.014 |
| No | 45 (9.8) | 33 (7.0) | 1.46 (0.93 - 2.29) | 0.095 |
| **COPD** | | | | |
| Yes | 30 (12.1) | 22 (8.8) | 1.39 (0.80 - 2.40) | 0.242 |
| No | 41 (9.9) | 25 (6.0) | 1.66 (1.01 - 2.73) | 0.043 |
| **Liver disease** | | | | |
| Yes | 21 (11.6) | 15 (8.2) | 1.42 (0.73 - 2.76) | 0.296 |
| No | 50 (10.5) | 26 (5.4) | 1.99 (1.24 - 3.19) | 0.004 |

*CKD stages 3–5 vs. Non-CKD*

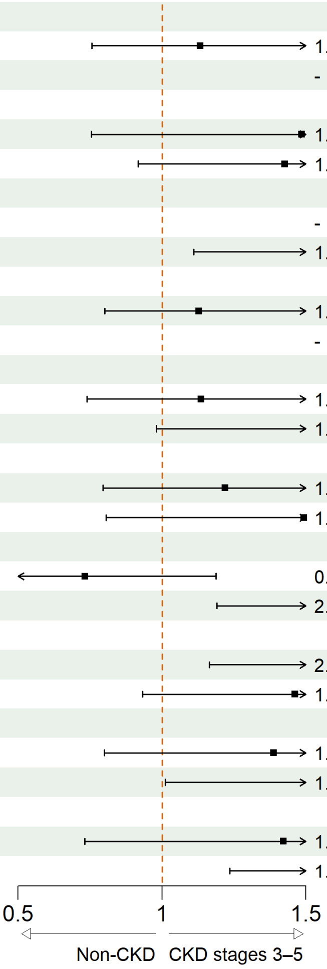


**Figure S5. Subgroup analysis for blood transfusion.** Forest plot of hazard ratios (HRs) with 95% CI for blood transfusion in matched esophageal cancer patients with CKD stages 3–5 versus those without CKD. Blood transfusion was defined by the transfusion procedure codes listed in Methods S1. HR > 1 indicates higher hazard in the CKD cohort.

## Figure S6.

**MACE**

| Subgroups | CKD stages 3–5 events (%) | Non-CKD events (%) | HR (95% CI) | P value |
|---|---|---|---|---|
| **Age (yr)** | | | | |
| ≥70 | 166 (53.2) | 152 (45.6) | 1.21 (0.97 - 1.50) | 0.094 |
| <70 | 37 (45.7) | 35 (46.1) | 1.02 (0.64 - 1.61) | 0.946 |
| **Nicotine** | | | | |
| Yes | 47 (59.5) | 50 (52.6) | 1.23 (0.82 - 1.82) | 0.318 |
| No | 152 (48.7) | 151 (45.6) | 1.11 (0.88 - 1.39) | 0.380 |
| **Alcohol** | | | | |
| Yes | 23 (51.1) | 25 (45.5) | 1.14 (0.65 - 2.01) | 0.652 |
| No | 176 (50.7) | 150 (41.6) | 1.25 (1.00 - 1.55) | 0.047 |
| **HTN** | | | | |
| Yes | 188 (51.8) | 179 (48.6) | 1.13 (0.92 - 1.38) | 0.255 |
| No | 17 (45.9) | 12 (32.4) | 1.50 (0.72 - 3.15) | 0.277 |
| **DM** | | | | |
| Yes | 108 (53.7) | 90 (44.3) | 1.27 (0.96 - 1.68) | 0.097 |
| No | 95 (48.5) | 89 (41.6) | 1.23 (0.92 - 1.65) | 0.155 |
| **IHD** | | | | |
| Yes | 108 (60.0) | 104 (53.3) | 1.11 (0.85 - 1.45) | 0.454 |
| No | 93 (43.5) | 96 (44.4) | 1.01 (0.76 - 1.35) | 0.927 |
| **CHF** | | | | |
| Yes | 56 (84.8) | 64 (87.7) | 0.92 (0.64 - 1.31) | 0.635 |
| No | 146 (44.4) | 130 (39.8) | 1.16 (0.91 - 1.47) | 0.223 |
| **CVD** | | | | |
| Yes | 42 (54.5) | 58 (63.7) | 0.80 (0.54 - 1.19) | 0.270 |
| No | 162 (51.1) | 141 (43.8) | 1.34 (1.07 - 1.68) | 0.011 |
| **COPD** | | | | |
| Yes | 71 (60.2) | 62 (55.9) | 1.10 (0.78 - 1.55) | 0.582 |
| No | 129 (47.4) | 128 (42.2) | 1.15 (0.90 - 1.46) | 0.274 |
| **Liver disease** | | | | |
| Yes | 59 (53.2) | 61 (54.0) | 0.95 (0.66 - 1.35) | 0.762 |
| No | 141 (50.4) | 150 (47.8) | 1.08 (0.86 - 1.36) | 0.499 |

*CKD stages 3–5 vs. Non-CKD*

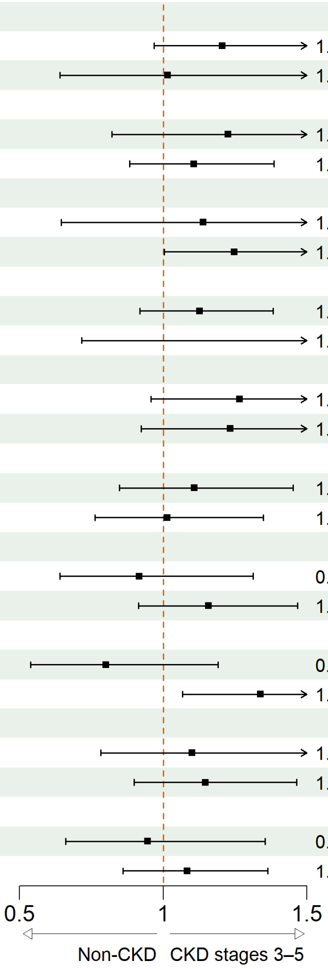


**Figure S6. Subgroup analysis for major adverse cardiovascular events.** Forest plot of hazard ratios (HRs) with 95% CI for major adverse cardiovascular events (MACEs) in matched esophageal cancer patients with CKD stages 3–5 versus those without CKD. MACEs were defined as acute myocardial infarction, subsequent myocardial infarction, intracerebral hemorrhage, cerebral infarction, other or unspecified nontraumatic intracranial hemorrhage, transient ischemic attack, all cause mortality, or heart failure. HR > 1 indicates higher hazard in the CKD cohort.
